# Robustness of performance during domain change in an esport: A study of within-expertise transfer

**DOI:** 10.1371/journal.pone.0295037

**Published:** 2023-12-07

**Authors:** Joe Thompson, Justin W. O’Camb, Robin C. A. Barrett, Scott Harrison, Mark R. Blair

**Affiliations:** 1 Department of Psychology, Douglas College, New Westminster, Canada; 2 Department of Psychology, Simon Fraser University, Burnaby, Canada; 3 Cognitive Science Program, Simon Fraser University, Burnaby, Canada; Edge Hill University, UNITED KINGDOM

## Abstract

Research on the transfer of skill from the circumstances in which it was learned to partially or completely novel tasks or situations is a foundational topic in the study of learning, memory, education, and expertise. A long history of transfer research has led to the conclusion that skill learning is generally domain specific. One important transfer problem occurs when a domain of expertise undergoes a fundamental shift, as when experts must adapt to changes in technology, rules, or professional practice. Here we examine skill maintenance in StarCraft 2, a video game of skills which undergoes frequent changes due to updates and includes a variety of gameplay options. Of particular interest are two competing predictions about how transfer will interact with expertise in this domain. The first approach emphasizes perceived similarity of the domains and predicts that skilled individuals will exhibit more favourable transfer than novices as these people will know enough to avoid processes, methods, and strategies which no longer apply after a domain change. The second emphasizes maximal adaptation to task constraints and predicts that experts will suffer the most during a domain change because of the loss of exploitable affordances. Neither approach did a good job explaining behaviour after the major game update called ‘StarCraft 2: Heart of the Swarm,’ perhaps because transfer was generally strong across all players. However, when examining transfer in the context of larger changes to gameplay, transfer seemed slightly better in more experienced players. The theoretical implications of this apparent interaction effect, and of the apparent resilience of more experienced StarCraft 2 players to transfer costs, are discussed.

## Introduction

An understanding of transfer is the holy grail of learning research. This is because, insofar as prior experience can be construed as a training task and future behaviour is construed as a transfer task, learning itself is a transfer effect [1]. A deep understanding of the mechanisms underlying transfer, would presumably allow for the prediction and control of learning, skill, and expertise. A good theory of learning, therefore, should be expected to predict and explain transfer effects (e.g., Thorndike & Woodworth [2]; for a review, see Adams [3]).

One of the early lessons from transfer research is that skill is domain-specific (e.g., Chase & Simon [4]). There are many examples where training tasks do not improve performance on a related task as much as one might like. Transfer costs can be found in sports [5, 6], infant locomotion [7], mathematics [8], luggage security screening tasks [9], spatial ability [10, 11], and education [12, 13].

Domain specificity has also been observed in research on transfer in video games. Tetris experts showed increased mental rotation ability, but only for Tetris or Tetris like shapes [10]. They didn’t show improvement in spatial ability tasks. When participants new to Tetris were given a 12-hour Tetris training program the domain specific improvements in mental rotation observed in experts were not observed in new trainees. Programs aimed at improving cognitive skills through digital games have repeatedly failed to show any real transfer effects [14–18]. In 2016, Lumos Labs, the company behind Lumosity, was made to pay $2 million [19] after their claims of far transfer between their games and other aspects of daily living were found to be unsupported by evidence. There are even occasions where transfer performance is worse after training [20]. Indeed, the conventional wisdom in skill research seems to be that far transfer, where training improves performance on a wide range of very different tasks, is rarer than expected and should never be assumed without empirical investigation [1].

Of course, real-world transfer effects must exist, or else learning would be impossible. For example, elite players of ball sports tend to have diverse sporting experiences [21], and some study techniques facilitate transfer better than others [22]. Nevertheless, predicting transfer remains highly challenging. While researchers can make real-world predictions by relying on the conventional wisdom that skill is generally domain specific, the conventional wisdom has little predictive value without an understanding of the moderators of transfer.

The present study examines the utility of two different theoretical frameworks which make competing predictions about transfer in the context of competitive esport video game play. The maximal adaptation approach construes expertise development as a process of increasing specialization, suggesting that transfer should become less effective with skill [23]. The similarity-based approach, in contrast, suggests that large transfer costs will be ameliorated in experts due to their educated perceptions about the similarity between test and transfer tasks [1]. We first test the prediction, common to all theories of transfer, that learning is generally domain-specific [1, 23]. We then examine competing predictions about how transfer effects will be impacted by expertise.

### Theoretical background

The present work defines transfer costs as a decrease in performance as participants transition from a training task to a test task [1]. The definition of ‘training’ and ‘test’ are intentionally left arbitrary, allowing transfer to be examined across many learning domains and across various theoretical frameworks. The definition also allows us to distinguish good transfer (where performance is maintained or improves from training tasks to test tasks), partial transfer (where performance is degraded but not to novice levels), non-transfer (where performance is degraded to novice levels), and negative transfer (where performance is degraded to below novice levels). A unified theory of transfer, therefore, would provide principles that could be used to predict how an animal will perform in a given situation (i.e., the transfer task) based on its set of learned experiences (i.e., the training task). Given the grandeur of this theoretical goal, it should be no surprise that no complete and unified theory of transfer is yet available. However, researchers have proposed theoretical frameworks and approaches to transfer which do nevertheless make predictions about when and where transfer will be observed. The present study will compare the predictions of two such approaches.

The similarity-based approach to transfer can be found in Kimball & Holyoak [1]. This approach maintains that successful transfer requires tasks which are objectively similar—in the sense that both tasks involve similar abilities—but also perceived as similar by participants. This recognition of similarity is thought to inspire the cognitive system to leverage skills from the training task in execution of the transfer task. We consider this to be a theoretical approach, rather than a well-specified theory, as researchers might disagree about how similarity should be defined [24]. However, this approach will lead to the prediction that transfer effects should be more favourable as participants become more experienced in the training task. This prediction stems from the fact that skilled participants, being more knowledgeable about the similarity between training and test tasks, will be able to best exploit whatever prior knowledge is relevant to the transfer task. In the context of expertise, this means that whatever opportunities of transfer are afforded by the training task will be identified and exploited by experts.

While Kimball & Holyoak [1] cache the similarity-based framework in theoretically neutral terms, more developed versions of the framework do exist. In Gick’s and Holyoak’s [25] theoretical framework, for example, transfer problems are put in terms of mental representations and rules for transforming these representations into procedures that produce behaviour. This framework follows Kimball’s & Holyoak’s [1] prediction that transfer is dependent on perceived similarity. In Gick’s and Holyoak’s framework, tasks would be perceived as similar when a common set of rules or representations would be deployed across a variety of different situations. In such cases, the cognitive system will attempt to apply its prior knowledge to a new situation, and this attempt will fail miserably if, despite perceived similarity, the transfer tasks has important structural differences.

The predictions of Gick’s & Holyoak’s [25] framework align with Kimball & Holyoak [1]. The cognitive systems of experts are likely, in the language of Gick & Holyoak to have rules that better match the structural regularities of the world making them less likely to attempt transfer when they should not, and this would lead to less negative transfer. We might also expect experts to have a great deal of positive transfer insofar as they have a highly developed network of many rules such that a solution to novel problems can be identified through the application of old knowledge. For example, if expert knowledge in physics is construed in terms of larger rule sets, then transfer of knowledge to novel physics problems might be expected insofar as a circuitous application of existing rules arrive at a solution. Similarly, experts may have more nuanced perceptions that represent a larger number of problem-relevant features, allowing them to use more resources in solving novel problems. So while the theories developed within Gick’s & Holyoak’s [25] framework may employ different theoretical language, the predictions of these theories align with the similarity-based approach. These approaches predict that transfer costs, when they can be found, should be ameliorated by expertise.

Not all frameworks would predict that the most experienced individuals will enjoy the most favourable transfer. Indeed, expertise is often construed as a maximal adaptation to task constraints [23]. In this case, it is reasonable to expect that changes to a domain of expertise will be especially damaging to experts, as their optimized performance is based on subtle task affordances that are overlooked by novices. This framework too, has sometimes been cached out in both representational and less-traditional terms. For example, representational descriptions of the maximal adaptation approach have been provided in the education literature (see, e.g., Gegnefurtner & Seppänen [13]). Under this conceptualization, the representations and schemas tied to expert performance are optimized to specific task conditions and cannot trivially be assimilated into other tasks.

Of course, the maximal adaptation approach does not need to be construed in such representational terms. For example, consider Adolph’s [26] finding that the avoidance capacities of young infants do not transfer from crawling to walking. Crawling-skilled but walking-noviciate babies tend to plunge over impossible gaps that they would not attempt to cross when crawling. Adolph views such transfer costs as due to the organization of control systems in infants. These motor systems are tied to posture in such a way that crawling skill cannot hope to transfer perfectly to walking. Adolph argues that highly general representations and rules, of the kind hypothesized in Gick’s [25] account of expert transfer, are not practical in the context of locomotor development, in part, because infant bodies change so rapidly.

Adults are, of course, not babies. Nevertheless, the learning of experts may well be tied to domain specifics that could interfere with some transfer performance. For example, while expert chess players have superior memory for chess positions, their ability is usually restricted to realistic chess positions and not truly random chess positions [4, 27]. We might expect, therefore, that a change to the rules of chess would seriously compromise chess memory performance if the rule change were to allow for new board positions that were previously impossible in a standard game of chess. From a perspective of maximal adaptation to task constraints, therefore, we might predict that novices will have better transfer than experts.

Regardless of how it is expressed, the maximal adaptation approach supposes that (a) expertise is a process of increasing optimization to the subtle particularities of a task environment and (b) transfer of skill to tasks outside of the learning environment will be laden with transfer costs. The additional optimization of an expert to a single learning environment is not likely to reduce transfer costs and, on the contrary, may make such costs worse. At the very least, the maximal adaptationist perspective does not hold out much hope for superior transfer of experts [13].

To test the predictive utility of these approaches one needs rich experience data comprising a large swath of the expertise continuum. The maximal adaptation framework, in particular, is designed for domains where learning occurs over hundreds or thousands of hours. Such task environments will be hard to construct in laboratory conditions as expertise takes thousands of hours to develop, and because cross-sectional comparisons of experts and novices can be misleading by only comparing snapshots of each player’s current level of expertise without showing each person’s individual development from novice to expert [28].

Here we examine transfer using real-world telemetry from skilled electronic sport (‘esport’) performance. First, StarCraft 2 is a domain of expertise, with full time professional players who have thousands of hours of practice. Furthermore, esports data collection can be performed automatically by participant computers, producing detailed measures of skilled performance across large sample sizes. This allows us to examine the impacts of domain change longitudinally and in the context of a player’s entire history of gameplay.

### The competitive esport StarCraft 2

StarCraft 2 is a real-time strategy game where the player manages economic and military resources of a civilization, while at the same time attempting to destroy the civilization of their opponent. The player must focus both on macro-level tasks, like managing their economy and expanding their bases, while also focusing on micro-level tasks like commanding groups of military units into battle.

Given the central role of Chess in the history of expertise research [4], it is useful to compare Chess and StarCraft 2. Both are strategy games which involve the competition between artificial armies following stable starting conditions. It is useful, however, to highlight two important differences between the two games.

First, StarCraft 2 requires a great deal of task switching. Unlike Chess, where players begin with a complete army, in StarCraft 2 players must switch between the collection of resources, the production of an army, the control of one’s own army, and discovering hidden information about the opponent. These shifts require directing attention to various areas of the interface, and switching the viewscreen to look at various places on the large scale map upon which the game is played (for an investigation of attention in StarCraft 2 see McColeman et al. [29]). Second, StarCraft 2 takes place in real-time rather than in turns like Chess, so there is a large incentive to issue commands as quickly as possible. Previous research has found that one of the most robust predictors of skill in StarCraft 2 is the speed at which players can act after allocating attention to a new area within the game [28]. In short, the game demands speedy performance while under high cognitive load.

Our research focuses on two transfer problems found in the video game StarCraft 2. First, on March 12, 2013, StarCraft 2 went through the ‘Heart of the Swarm’ expansion. The Heart of the Swarm expansion was a particularly large change as it introduced seven new usable game units, each with unique appearances and abilities that had important gameplay implications. This change enabled new kinds of tactics and made many strategies less effective. Any player who purchased the Heart of the Swarm expansion had to integrate these units into their play. Even if they themselves were not using the new units, they would have to learn to counter the new dynamics imposed by the new units if their opponents opted to use them.

Different theoretical frameworks disagree as to how the impact of the expansion on performance should interact with prior expertise. Kimball’s & Holyoak’s [1] similarity-based framework would predict that more experienced participants will have more detailed and accurate knowledge of the differences between pre and post-expansion gameplay, and therefore also expect the magnitude of transfer effects to become more favourable with prior experience [1]. However, a maximal-adaptation approach [23] would lead one to expect less favourable transfer with prior experience insofar as only experts are skilled enough to rely on subtle contingencies that were disturbed by the release of Heart of the Swarm.

A second transfer problem that emerges in the context of StarCraft 2 comes from choices in gameplay. Players are given a wide range of latitude in how they approach the game. Unlike in Chess, where the available chess pieces remain the same for all players, StarCraft 2 players must choose a ‘Race’ at the beginning of each match, each of which is associated with an entirely new set of pieces. Each piece is associated with its own advantages and disadvantages, and many pieces are associated with unique special abilities that alter the basic mechanics of gameplay. There are also more fundamental differences between the StarCraft 2 races. Unlike Chess, where pieces are granted at the beginning of the game, StarCraft 2 players must manage economic considerations while developing their civilization, first collecting resources and building prerequisite buildings prior to being able to produce any military units. Some races, for example, may produce their units through a series of disjointed structures, while another must ‘grow’ their pieces through a central structure called a hive. This requires that every action of a player is then coloured in some way by their choice of race, meaning that the perfect transfer of skill between races seems unlikely given the domain specificity of expertise.

Given the large change associated with changing race and the general finding that transfer is difficult, we once again predict a general loss in performance. As with the prior analysis, experienced players who switch Races enjoy a superior knowledge of the game in general, suggesting more favourable transfer from a similarity-based framework. In contrast, an emphasis on maximal-adaptation to task constraints suggests that the major change in gameplay associated with a change of race would be especially punishing to those who were highly adapted to the task in their preferred race.

## Method

### Data collection

Participants were recruited from internet gaming communities, and 124 StarCraft 2 players filled out a survey and, collectively, submitted 164,001 game replay files for analysis. The collected self-report survey was, for the present project, consulted only to determine each player’s years of experience with the original StarCraft game, on which StarCraft 2 was based (see Analysis 3 for details). The primary measures are performance-based measures found in replay files collected from each player. The collected replay files are automatically generated by the game itself, and are encoded such that they are difficult to modify. They include the information the game engine requires to replay game in full. This file, once parsed, provides us with timestamped lists of every action players took during each game, producing the files from which our performance data is calculated. The average game lasted roughly 15 minutes.

To be included in our study, games needed to be verifiable StarCraft 2 games played by the survey respondent (see S1 Text for details about the verification process). After this initial exclusion, 109 players and 117,978 StarCraft 2 games remained for analysis. StarCraft 2 is a complex game with several different game modes. Some of these game modes, such as games between two *teams* of players, are relatively rare. We further restricted our analysis to games that contained two human players that were randomly matched against each other using the game developers automated matchmaking system. This helps to ensure that players were matched against motivated opponents of roughly equal skill. This left us with 107 players (103 males; 2 females; 1 other; 1 unknown) and 81,655 replays, which is equivalent to roughly 20,000 hours of second-by-second performance data. Ages of these players range from 16–41, with a mean age of 24.65, and a standard deviation of 5.28 years.

### Definition of experience

The definition of experience is challenging in the complex environments such as StarCraft 2 [30]. This is because StarCraft 2 has several different game modes, including team games and games with opponents selected by the player rather than opponents selected by the match-making system. This issue is especially challenging when one is investigating transfer between game modes because, if transfer were poor, then experience would be better measured by more restrictive definitions that are specific to the number of games a player has played in a particular game mode. If transfer effects are strong, then more permissive definitions, which include a wider range of game modes, might be better. Given that transfer effects can be unpredictable, we begin with a more permissive definition but plan to rerun analyses with more restrictive definitions as necessary. We initially measure a player’s current experience, therefore, against the number of verified StarCraft 2 games in our database (117,978 such games are in our database) which they have participated in up until that date. To ensure results are not artifacts of our definition of experience, we sometimes rerun analyses with a stricter definition of experience as one-versus-one games with an opponent found using the match-making system (81,655 such games are in our database; (see S1 Text for details about the verification process).

### Dependent variable: League-equivalent performance

Our primary performance measure for our analysis is League-equivalent Performance and is derived from looking-doing latency, which is defined as the mean latency of actions that immediately follow a shift in attention within StarCraft 2. Looking-doing latencies are the first component of Perception-Action Cycles of a StarCraft 2 game, a cycle which includes fixating the game screen on a new location, acting, and shifting the screen away [31]. This measure is of interest to psychologists as it is inspired by the perception-action cycles that make up real-world tasks [32]. Prior work has found that looking-doing latency is a robust predictor of StarCraft 2 skill [28], exhibits patterns of learning-related changes in information-access behaviours consistent with laboratory eye-tracking research [29], and shows age-related changes [33].

There are two factors that make using raw looking-doing latency less desirable for the present study. First, looking doing latency is associated with a player’s game ‘race’. Given that the mechanics of gameplay differ significantly between races, it is not surprising that previous cross-sectional datasets revealed differences in performance speed by race [34]. Second, looking doing latency numbers are a timing measure and so interpreting transfer results in terms of the total continuum of skilled performance is more difficult than necessary. In the present work, we rescale looking-doing latencies into a measure of performance with the aim of addressing these two problems.

Using the cross-sectional dataset of 3,307 games from Thompson, Blair, & Henrey [28], we fit the relationship between Looking-Doing Latency and League, taking into consideration game race. This analysis revealed that Zerg players have lower Looking-Doing-Latencies for any given league than the other two races, and that the relationship between Looking-Doing Latency and League was clearly linear. There was no apparent interaction between league, race, and looking doing latency. We therefore refit the models, one for Zerg players, and the other combining Protoss and Terran players, and used these models to transform Looking-Doing Latency into a league-based continuous scale for the present data that we call League-equivalent Performance. A score of 1 equates to a Looking-Doing Latency of the weakest level of online play: Bronze-League. A League-equivalent Performance score of 8 equates to professional level play for that given race.

Performance data spanned a wide range of skill levels. Roughly 25% of our games included performance comparable to the lowest levels of skill (League-equivalent performance <1.5 = 7.4%; <2.5 = 13.5%; <3.5 = 23.3%), and a little more than a third of our games came from the highest levels (League-equivalent performance >5.5 = 37.3%).

## Results

### Analysis 1: Transfer of performance between two major versions of StarCraft 2

In analysis 1 we compared player performance (using the league-equivalent performance measure for every game for every player) from before and after the release of the StarCraft 2: Heart of the Swarm expansion and examine whether transfer effects might vary by prior skill. To make full use of the dataset, we constructed two linear models for each player that predicted League-equivalent performance based on the number of games played—the first model used performance data from games prior to the expansion, and the second used data from games after the expansion. We then compared the model predictions for the first game played after the expansion. This allowed us to generate a prediction-difference transfer estimate for each player and to conduct one-sample t-test to establish whether the earliest StarCraft 2: Heart of the Swarm performance was in keeping with the skilled performance associated with StarCraft 2: Wings of Liberty.

We hypothesized a non-zero prediction-difference transfer estimate, i.e., we expected the performance estimate from before the expansion to exceed the performance estimate from after, as the domain specificity of expertise implies that performance should suffer after such a major change. We then tested the predictions of the similarity-based and the maximal-adaptation based frameworks by examining whether transfer would be favourable for more experienced players. This was examined with a significance test of the Pearson correlation coefficient between the number of games played before the expansion and the prediction-difference transfer estimate. Analysis 1 revolves around the comparison of linear models before and after the release of the Heart of the Swarm expansion. Players need to have played at least 20 games before and 20 games after the expansion to be included in this analysis, leaving us with a sample size of 32 players and 19,312 games.

**Fig 1:**
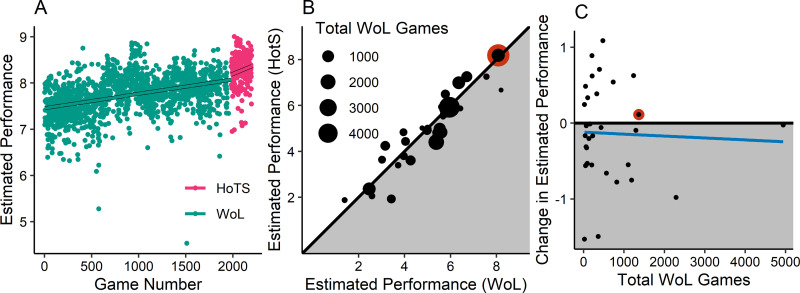
Results from expansion analysis. (A) League-equivalent Performance for the games of participant #78. Green dots are games of the original SC2 Wings of liberty (WoL), and pink dots are games of the Heart of the Swarm expansion (HoTS). Lines for each game type represent best fitting model of performance by game number. (B) Estimated performance for the next game using the original SC2 (WoL) model plotted against the estimated performance for the initial game using the HoTS expansion model. The size of each point reflects the total number of games used to estimate that player’s performance. The highlighted point is the estimate for the same player as panel A. Data in the shaded region show loss of performance when players transferred expansions. (C) Estimated transfer effect of the switch to the expansion (estimated performance WoL—HotS) plotted against total experience at the time of transfer. The highlighted point is the estimate for player #78, from panel A. Data in the shaded region show loss of performance when transferring.

The data of a single participant (#78 in our dataset) is shown in Fig 1A, with league-equivalent performance plotted for each successive game. The change from the original version of SC2 to the expansion version is indicated by a change in the dot colour. The best fitting linear models for each expansion are highlighted in black for visibility. A descriptive comparison of the model predictions is summarized in Fig 1B, where the proximity of data points to the reference line suggests strong transfer. Predictions of early Heart of the Swarm Performance were similar regardless of whether predictions were generated by pre-expansion or post-expansion data. Fig 1C, which depicts the relationship between experience at the time of transfer and prediction-transfer-estimates, reveals little evidence of a transfer-experience interaction.

We began the formal analysis with a one-sample t-test on prediction-difference transfer estimates. Sample standard deviations were low. This is not surprising given that the typical model prediction was based on 1,081 of games per player, the majority of which contain 253 first-action-latencies. Tight standard deviations lead to a respectable power to detect 1 league-equivalent performance difference (α = .05, 1- β ~99%, N = 32, δ = 1). Nevertheless, we failed to find significant prediction-differences between the models (t(31) = -1.193, p = 0.242, 95% CI: -0.36, 0.095). In short, performance in the first games of Heart of the Swarm performance did not seem to differ markedly from Wings of Liberty performance.

We also conducted a correlation test on the Pearson correlation between the number of games played before the Heart of the Swarm expansion and the prediction-difference transfer estimate. We failed to find significant prediction-differences between the models (r = -.038, t(30) = -.209, p = 0.835, 95% CI: -0.38, 0.314), though power was only sufficient to detect a large effect size (α = .05, 1- β = ~85%, N = 32, δ = 0.5). Two points were influential, but excluding these points had no effect on the results. To ensure that these results were not due to an inclusive definition of experience (which includes many game modes), we also reran the aforementioned analyses using a more restrictive definition of experience that included only 1v1 games created using the match-making system. The choice of definition had no discernable impact on the pattern of results. In summary, analysis 1 revealed evidence of strong transfer of performance following a major game update, and little evidence that transfer effects differ by prior experience.

### Analysis 2: Transfer between two different ‘races’ in StarCraft 2

Analysis 1 discovered strong transfer between the two different expansions of StarCraft 2. This is surprising given the domain specificity of expertise. In Analysis 2 we investigate even more substantive changes to gameplay.

Unlike chess, where both players always control pieces that are equivalent sets of units, players in StarCraft 2 often have their own units corresponding to which “race” a player chooses to command at the beginning of each match. Each race is equipped with distinct buildings, units, and game play mechanics, all working together to enable a diverse set of unique tactical possibilities which must be learned to gain proficiency in that race. Due to the significant differences in the tactics, strategies, and timings that result from differences in how each race functions, players often choose one race to play as their as their dominant race, as playing with another race would require significant additional learning. As such, if a player chooses to use a race other than their dominant race in any given match, the difference in required skills for that race creates a transfer problem for that individual game. Given that a change of race implies changes to so many aspects to gameplay, we expect weaker transfer of performance than in Analysis 1.

Analysis 2 brought new data analytic challenges. Unlike Analysis 1, which focused on a single point in time (i.e. the release of StarCraft 2: Heart of the Swarm), players choose their race at the beginning of each match, and could therefore change what race they play as at any point in time. Consequently, a more sophisticated analysis using mixed effects models was required. Analysis 2 focuses on two independent variables, a training/transfer variable that differentiates the games using the players dominant race (the one they use most often) from the transfer games where players are using a different race (often called “playing off-race”), and an experience variable that quantifies that players amount of experience at the time of play. A model without the training/transfer variable, which makes predictions solely based on general experience, effectively assumes perfect transfer: predictions are not adjusted if a player chooses to play their non-dominant race. Our first research question asks whether a model that includes the training/transfer variable predict performance (once again using our league-equivalent performance measure derived from looking-doing-latency) better than a model without it. Our second, and more central research question, is investigated by adding an interaction term to our model to determine if players with more experience have more favourable transfer performance than players with less experience. Interestingly, players exhibited a strong preference for a dominant race, and many players avoid off-race games. To be included in analysis 2, players needed to play at least 30 off-race games and at least 50 games overall. One player was dropped for having an even distribution of races. This left us with 18 players and 14,625 games for our analysis. The fact that so few players qualified for this analysis is evidence of participant reluctance to play off-race.

**Fig 2:**
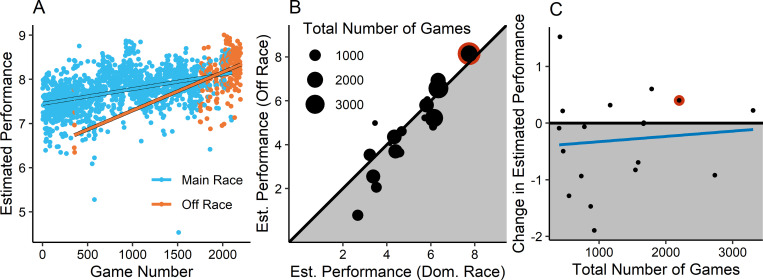
Results for race analysis. (A) League-equivalent Performance for the games of participant #78. Blue dots are games of the players main race, and orange dots are off race games. Lines for each game type represent best fitting model of performance by game number. (B) Estimated performance for the model of Dominant Race games plotted against the estimated performance using the model of Off Race games. The size of each point reflects the total number of games used to estimate that player’s performance. The highlighted point is the estimate for player #78, from panel A. Data in the shaded region show loss of performance when playing Off Race. (C) Estimated transfer effect of the switch to Off Race (Dominant Race—Off Race) plotted against total experience. The highlighted point is the estimate for player #78, from panel A. Data in the shaded region show loss of performance when playing Off Race.

The data of a single participant (#78 in our dataset) is shown in Fig 2A, with league-equivalent performance plotted for each successive game. Dominant race games and off race games are in differing dot colours. The best fitting linear models for each expansion are highlighted in black for visibility. A descriptive summary of our results can be found in Fig 2B. The proximity of dominant race performance and off race performance to the reference line, is again suggestive of strong transfer. Fig 2C shows what may be a weak transfer-experience interaction, as less experienced players seem to be suffering greater drops in performance when they play off-race.

Although Analysis 1 only had power to detect a large interaction effect between training and experience, Analysis 2 has power to detect considerably smaller effects. To assess the power to detect an interaction effect, we used the simr package in R [35]. A range of effect sizes were selected a priori. However, simulating power for mixed effects models requires specification of a covariance structure, which is not supplied by psychological theory or the StarCraft 2 community. We therefore calculated power in a post-hoc fashion, by using the observed covariance structures derived from Analysis 2 below. We defined a small interaction effect as a reduced transfer cost of one tenth of a league of skill after 1,000 games of experience. Simulation results suggested that we have respectable power even for detection of small effects (α = 0.05; 1- β = 94.5%, 95% CI [92.9, 95.83], δ = .01).

We regressed the number of competitive games experienced onto League-equivalent Performance in a mixed effect model, using a random intercept for player using R [36], with the lme4 package by Bates, Maechler & Bolker [37]. We compared this model to more complex models built with all prior significant effects; each of the nested models were compared using likelihood ratio tests. No games were found to be overly influential (D_i_<1).

The first model was League-equivalent Performance predicted by number of competitive games with a random intercept for each player. Each subsequently added effect was found to significantly increase the fit of the model: a random slope for each player (χ2 = 2201.9, p<0.001), our dichotomous training/transfer variable (χ2 = 24.911, p<0.001) (Beta = 0.26, 95% CI [0.179, 0.339]), and an interaction effect between number of competitive games and dominant or off-race games (χ2 = 16.666, p<0.001) (Beta = -0.012, 95% CI [-0.017, -0.006]). Our observed effect size was for this interaction was that those playing off-race preserved an additional 0.12 leagues for every 1,000 games of experience possessed. Given that our best estimate of the transfer cost of playing off-race is about 0.26 leagues of skill, we expect it would typically take about 2,000 games of experience to eliminate the transfer costs associated with playing off race. This interaction effect is small given that 1,000 games seems to have a main effect of roughly 1.3 leagues of overall improvement.

One challenge with this analysis was the definition of player experience. StarCraft 2 has a variety of game modes, and so it is sometimes unclear what sort of experience should be counted as prior experience. Following Thompson [30] experience was defined to include both 1 versus 1 and team games. In other words, we define experience as any game of StarCraft 2 that passed our initial exclusion criteria (~118,000 games), regardless of game mode. Given that the topic of the present study was transfer, we also reran analysis 2 with a more restrictive definition. In this case, we restricted experience to games with exactly one participant and one opponent using the games match-making system. Using this alternate definition of experience led to worse model fits overall. This fact is consistent with the generally strong transfer we see across variations in the game, that is, team games seem like valid examples of experience and excluding them weakens the model fits. Regardless, in these new analyses, models were improved by the addition of a random slope (χ2 = 1896.5, p<0.001) and a dichotomous training/transfer variable (χ2 = 24.4, p<0.001), but, unlike the initial version, the interaction term did not significantly improve models (χ2 = 0.282, p = 0.596). Our best interpretation of this situation is that, while the superior models support the predicted finding that experience modulates transfer, this finding is neither strong, nor especially robust.

### Analysis 3: Transfer between StarCraft 1 and StarCraft 2

In Analysis 2 we again found strong transfer, defying the general expectation of domain specific performance. Given the presence of strong transfer across the three StarCraft 2 races, a transition involving entirely different units and mechanics, one might wonder what sort of intra-domain transfer could have a meaningful impact on performance. Any player of StarCraft 2 would recognize that it was modelled after its predecessor. Both games have dedicated full-time professional players and both are recognized as Esports in the scientific literature [38]. The games are also similar enough to enjoy some overlap in their communities, as evidenced by the fact that Esport websites covering StarCraft 2 often report on StarCraft 1 as well. Furthermore, there are individuals capable of professional play in both games [39, 40]. In short, to two games are different enough to be considered separate domains but similar enough for some transfer to be possible.

To gauge farther transfer, our final analysis examined whether the first fifty games of StarCraft 2 performance can be predicted by self-reported experience in StarCraft 1, the game to which StarCraft 2 is a sequel. Self-reports of experience were obtained by asking participants the year in which they began and ended playing StarCraft 1 and calculating the difference. Our change from the preferred direct measure of performance in Analyses 1 and 2, to a measure of self-reported experience was out of necessity. Not only are StarCraft 1 replays unavailable for most players, but even if the records could be collected, the data contained in StarCraft 1 replays is more limited and does not allow for the calculation of looking-doing latency or league-equivalent performance.

While the previous two analyses involved a comparison of performance based on game recordings, the analogous data are not available for StarCraft 1. In this final analysis we look at whether experience in StarCraft 1 is predictive of skill in StarCraft 2 by adding self-reported years of experience with StarCraft 1 to our best model of the first fifty games of StarCraft 2 performance and evaluate this updated model with a likelihood ratio test.

**Fig 3:**
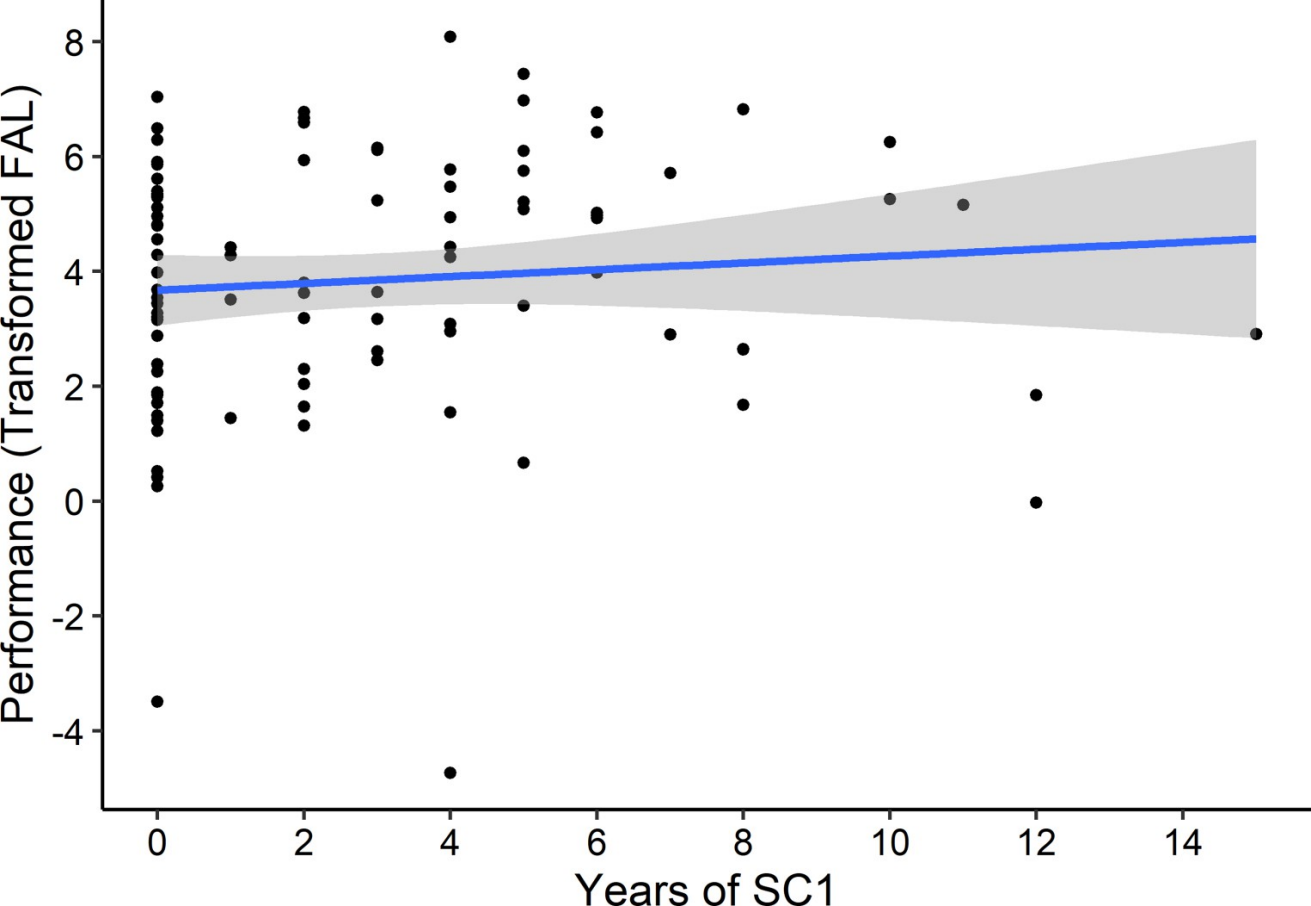
Results for StarCraft 1 analysis. Mean League-equivalent performance by self-reported years of SC1 experience for each player’s first 50 SC2 games. Statistical inference was based on mixed effects models that are not depicted in this figure. See text for details.

Participants in Analysis 3 satisfied our general exclusion criteria and played at least 3 competitive 1-versus-1 matches in their first 25 games. A total of 97 players satisfied these criteria, and 93 also reported when they began and ended their play of StarCraft 1. A depiction of the observed relationship between self-reported StarCraft experience and StarCraft 2 performance are summarized in Fig 3.

We found no significant relationship between the number of years of StarCraft 1 experience and the first three competitive games of StarCraft 2 performance (r = 0.157, t(30) = 1.51, df = 91, p = 0.136, 95% CI: -0.05, 0.350), despite respectable power (α = .05, 1- β = ~99%, N = 93, δ = 0.5). There is little evidence of an association between StarCraft 1 experience and early StarCraft 2 performance.

## Discussion

Transfer effects are hard to predict. Perhaps not surprisingly, observations of transfer have played an important role in falsifying theories of learning [2, 3]. The topic of transfer continues to be a central to theories of learning, expertise, motor skills and categorization. In the present study we investigated transfer effects in a real time strategy game using replay files that document performance, often over hundreds of hours of experience. This research method has the advantage of allowing us to assess transfer using identical performance measures during training and transfer. Further, we have a sense of how observed performance levels fit within the spectrum of expertise on a given task, and can thus assess transfer losses in relation to total skill in the domain. This allows us a unique opportunity to evaluate predictions about transfer in complex task domains. The specific predictions we focused on here are in relation to one potential moderating factor of transfer costs: prior experience. Frameworks predicting transfer based on perceived task-similarity [1] have suggested that more experienced individuals should achieve better transfer, while those frameworks that construe experts as maximally adapted to a single task [23] suggest the opposite.

We documented transfer costs when players played a race other than their dominant one (Analysis 2), and these costs seemed slightly larger for novices. An additional 1,000 games of experience (which corresponds to roughly 250 hours of play) was associated with a reduced transfer cost of 0.12 leagues when players changed gameplay race, and our best models suggest that 2,000 games would be enough to ameliorate most of the transfer costs of playing off-race. Our general pattern of results therefore fit best with the similarity-based framework described in [1]. Of course, this apparent theoretical victory is diminished by a weak, and not particularly robust, interaction effect between transfer cost and experience.

In contrast, it seems like StarCraft 2 performance does not fit well with the concept of maximal adaptation. Previous research provided evidence against one application of the maximal adaptation framework to motor chunking in StarCraft 2. Specifically, prior research examined the idea that the impressive speeds of StarCraft 2 players should be attributable to a small number of extremely specialized, and overlearned, motor-chunks [27]. This chunk-centric perspective suggests that expert performance is due to highly specific, and non-transferrable action sequences that become mastered throughout experience. However, despite the efforts of prior work [34] to identify chunks and measure their impact on performance, better StarCraft 2 players were faster regardless of the action sequences being performed. The present findings are more general and suggest that the players who are expected to be the most specialized (i.e., experts) were, contrary to the maximal adaptation framework, the most resilient to transfer costs.

One of the puzzles of the present data are the surprisingly small transfer costs observed across all participants. Analysis 1 showed remarkable transfer. Predictions of post-expansion performance were virtually the same, regardless of whether the model was built from pre or post-expansion performance. There were consequently no transfer costs for experience to ameliorate.

The stronger transfer costs for non-dominant race play, as described in Analysis 2, were not unexpected given that changing race impacts more pieces and gameplay mechanics. Nevertheless, effect sizes were surprisingly weak. Playing a non-dominant race was associated with about a quarter of a league in terms of performance. This is quite small relative to the typical level of league-equivalent performance in our sample, which was about 5. When taken into consideration alongside Analysis 3, our results suggest impressive transfer within the domain of StarCraft 2, but not between StarCraft 2 and its predecessor.

One explanation for generally impressive transfer is that the novices in our sample are already too knowledgeable about StarCraft 2 to suffer a within-domain transfer cost. This explanation, while technically consistent with the similarity-based approach, would undercut its utility as an approach to predicting transfer. The below-average players in our sample each have around 250 hours of experience and includes players with a league equivalent performance of 3.8, placing them close to what is known as ‘platinum league’ on a scale that, including professional play, contains 8 levels of skill in total. Even this level of performance would not be considered exceptional, or even intermediate, in the StarCraft 2 community. Importantly, we do not claim our results are inconsistent with this possibility. Instead, given the ubiquity of transfer costs in the learning literature, we think it implausible that the skill of our novices *alone* could explain the weak or absent transfer costs that we observe here. If novitiate levels of skill were sufficient to ameliorate such costs, domain specificity should be very rare in the natural world. Instead, we find it more likely that, if our StarCraft 2 novices are somehow using their knowledge to circumvent transfer costs, then there must be something unique to this domain which makes such transfer more impressive than usual.

A second, more plausible, explanation for our results is that there is some feature of StarCraft 2 that instills resilience in player performance. One such feature may be the changing task environment of esports. Most domains are stable, like chess, with changes being both rare, and minor. In esports, software developers are constantly changing the parameters and rules of the game to ensure competitive play that is entertaining for viewers. Indeed, esports communities have even developed a common language to describe these changes. For example, when companies ‘balance’ the game, some methods of play receive an advantage or ‘buff,’ while others become less effective, or are ‘nerfed’ [41], and the over pattern of strong strategies is called the ‘meta’. It is plausible that playing against a backdrop of constant change in the task has yielded impressive protection against the sort of transfer costs examined in Analysis 1.

A final, and related, possibility is that player decision making is impacting their resilience to transfer costs. StarCraft 2 players have always been able to choose their own race and therefore have control over this aspect of their task environment. Furthermore, earlier studies of age-related change in StarCraft 2 suggested that older players, while slower, can adapt their play style to remain competitive [33]. In Analysis 2 of the present study, we noticed that many players were clearly avoiding certain races (see exclusion criteria for analysis 2) and those who did play off-race exhibited transfer costs that were ameliorated by experience. Our evidence therefore fits with the picture of a cognitive system that, in its attempt to win games and improve, is attempting to dynamically manage task difficulty and transfer effects by exerting control over the task environment (e.g., by adjusting race, strategy, or play-style).

There are some important limitations of our work. First, since data was collected based on files donated by players, it is not possible to verify that players submitted all of the digital records associated with their gameplay. Given that records were collected automatically by the game, it seems unlikely that players would go to the effort selectively donate unrepresentative games, such as games where they performed well. However, it is possible that a player could, for example, have lost a large batch of their noviciate performance due to a hard drive failure. This sort of data, which we would consider missing at random, could yield misleading learning curves for some players. Secondly, it is important to recognize that the study of transfer needs to be separated from the question of generalizability. While we study transfer between different kinds of gameplay within the game of StarCraft 2, we make no claims about whether similarly powerful transfer effects would be observed within other games or domains. It is possible that there is something special about StarCraft 2 task environment that produces strong transfer effects. It is also possible that *strong within-domain transfer*, is common but undetected by older research methods. In 1973, Newell [42] advocated for the analysis of a complex task as a way forward for cognitive psychology. Such an endeavour requires integrating the diaspora of findings in cognitive science into a larger theoretical network that can be evaluated for its capacity to predict and explain behaviour. Our results confirm that such a unified framework is not yet available. However, recent advances in our ability to collect data on complex tasks through telemetry [31, 43–45] provides cognitive science with the opportunity to identify new phenomena to be explained. We hope this incentivizes comprehensive theoretical approaches with the capacity to make clear and nuanced predictions about the presence and magnitude of transfer costs.

## Supporting information

S1 Text Supplementary definitions. (PDF)
