# Correction: Robustness of performance during domain change in an esport: A study of within-expertise transfer

**DOI:** 10.1371/journal.pone.0317465

**Published:** 2025-01-09

**Authors:** Joe Thompson, Justin W. O’Camb, Robin C. A. Barrett, Scott Harrison, Mark R. Blair

The first author, Joe Thompson, is incorrectly noted as the corresponding author. The correct corresponding author is Mark R. Blair. Mark R. Blair’s email address is: mblair@sfu.ca.
