# Supplementary material for: Robustness of performance during domain change in an esport: A study of within-expertise transfer
Source: PLoS One. 2023 Dec 7;18(12):e0295037. doi: 10.1371/journal.pone.0295037 (PMC10703280; doi:10.1371/journal.pone.0295037)
Supplement: S1 Text — (PDF) [file pone.0295037.s001.pdf]

# Supplementary materials/definitions

## Background on StarCraft 2: Heart of the Swarm

Heart of the Swarm is the second game in a trilogy of games that make up the totality of the game Starcraft 2. Each version had a different single player campaign, each new campaign focusing the single player game on a different race. The third game in the trilogy was called Legacy of the Void, it was released in 2015. All our data was collected before the release of Legacy of the Void, so no games from the third game in the series were included in our dataset. We refer to Heart of the Swarm as an upgrade of Wings of Liberty, but it is technically more than that, it is a new game, a sequel. It includes new units to to the online part of the game on top of the new single player campaign. This online component is what we are discussing when we talk about the changes from Wings of Liberty to Heart of the Swarm. We do not collect games from the single player campaign.

The core units from Wings of Liberty remained in this updated version of the game, though these units were also sometimes substantially modified, for example a unit from Wings of Liberty can be transformed, using an in-game upgrade mechanism, into a new stronger version of the same unit in Heart of the Swarm effectively making it a new unit. Depending on race, two or three new units were added to each race in Heart of the Swarm. These were added onto the fifteen to seventeen units, depending on race, which the player already had access to in Wings of Liberty. Game developers design these new units with the specific intent to alter the way combat unfolds in the game and to make new strategies viable. The units completely change the game dynamics and therefore requiring a change to the set of strategies used by any given player and their opponent. In the creation of Heart of the Swarm, the new units were carefully compared to existing units, and both the new units and existing units were modified together in such a way so as each race is equally balanced so that no race is unfairly stronger than another.

The player, once buying and downloading Heart of the Swarm which then updates Wings of Liberty, cannot go back to playing the game they were playing before the update. They must adapt to the new

dynamics, and given the novelty of new units will likely incorporate them into new strategies and styles of play. This gives us a unique and specific opportunity to study learning transfer. Given every player is essentially playing a novel, and modified version of the game they were previously playing we have a cutoff point where the game was once played under specific dynamics, and then at the cutoff point, the release and download of Heart of the Swarm, changes the totality of the game dynamics into a new version of the game they were previously playing where new strategies are now required to be a successful player.

# Definition of Any_XP/Performance

Of the initial 164,001 file submissions, a number of replay files could not be verified as authentic. To be used in our dataset, a replay file would need to pass the following tests:

- The game needs to have no duplicates in the database
- Performance data needs to be traceable back to a unique, successfully parsed replay file and survey submission
- At least one player in each game needed to be a survey respondent
- The game was donated by a unique survey respondent
- The game had a unique player and game identifier

Games which pass these minimal criteria (n=117,978), and were therefore considered genuine games of StarCraft 2, were considered part of a players background experience (and reflected in the variable ‘Any_XP’).

Importantly, StarCraft 2 also contains a number of different game modes. Players can play in other races, play team games (e.g., 2v2, 3v3), or even play custom games against non-random opponents. The present project treats such games as experience, but does not wish to conflate performance in such games with performance in definitive 1v1 play

against random skill-matched opponents. We therefore examined performance data, including looking-doing latencies, from 81,655 games and 107 players.

While performance data was only examined in 1v1 play against random skill-matched opponents, our definition of experience was more inclusive. When recording a players prior experience in the variable 'Any_XP', we used data from all 117,978 games that passed our minimal exclusion criteria.
